# Neurodegenerative spliceosomopathies

**DOI:** 10.3389/fcell.2026.1787859

**Published:** 2026-05-28

**Authors:** A. Mavillonio, D. Rizzini, S. Detassis, M. A. Denti

**Affiliations:** Laboratory of RNA Biology and Biotechnology, Department of Cellular, Computational and Integrative Biology (CIBIO), University of Trento, Trento, Italy

**Keywords:** amyotrophic lateral sclerosis, retinitis pigmentosa, RNA splicing, snRNAs, spinal muscular atrophy, spliceosome

## Abstract

Spliceosomal syndromes are a group of disorders caused by pathogenic variants in core spliceosomal RNAs or proteins, leading to defective pre-mRNA splicing and tissue-specific disease vulnerability. Although the spliceosome is ubiquitously expressed, its dysfunction preferentially affects highly splicing-dependent tissues such as the retina and the nervous system. This mini-review focuses on neurodegenerative spliceosomopathies, including spinal muscular atrophy, amyotrophic lateral sclerosis, and retinitis pigmentosa, highlighting how alterations in snRNP biogenesis, spliceosome assembly, and splicing fidelity drive neuronal and photoreceptor degeneration. We discuss shared and distinct molecular mechanisms, unresolved questions on tissue specificity, and emerging therapeutic strategies targeting RNA splicing.

## 1 Introduction

Spliceosomal syndromes are disorders caused by pathogenic variants in genes encoding core spliceosomal RNAs or proteins, leading to defective pre-mRNA splicing and marked tissue-specific vulnerability. These conditions preferentially affect the retina, craniofacial structures, bone marrow, limbs, and spinal cord, giving rise to clinically diverse diseases such as retinitis pigmentosa (RP), craniofacial malformations, spinal muscular atrophy (SMA), and amyotrophic lateral sclerosis (ALS) (Will and Lührmann, 2011). Importantly, not all disorders associated with aberrant splicing are classified as spliceosomopathies, which by definition require mutations in core spliceosomal components (Deutsch et al., 2025).

**FIGURE 1:**
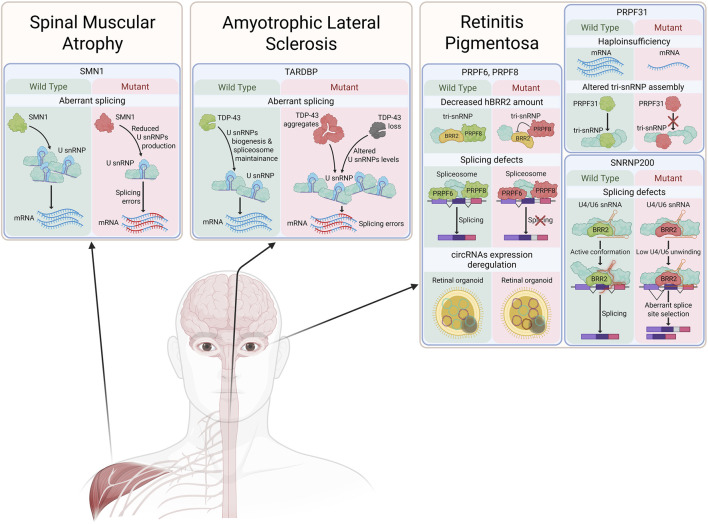
Spliceosomal dysfunctions in neurodegeneration. Genetic alterations affecting core splicing factors disrupt key spliceosome functions - such as snRNP biogenesis, assembly, and splice site selection—leading to widespread RNA processing defects in neurological and neuromuscular disorders.

Pre-mRNA splicing is catalyzed by the spliceosome, a large ribonucleoprotein complex composed of small nuclear ribonucleoproteins (snRNPs) and associated proteins that mediate intron removal and exon ligation (Will and Lührmann, 2011; Wilkinson et al., 2020; Zhang et al., 2024). While constitutive splicing ensures transcript maturation, alternative splicing expands proteomic diversity and enables precise spatio-temporal regulation of gene expression. In the nervous system, alternative splicing is essential for neuronal development, identity, and network function; its disruption impairs neuronal homeostasis and contributes to neurodegenerative disease (Nik and Bowman, 2019). Since available literature on spliceosomopathies predominantly investigate neurodevelopmental disorders and is already extensively covered, this review focuses on neurodegenerative spliceosomopathies with major incidence (Cooper et al., 2009; Scotti and Swanson, 2016), arising from defects in spliceosome machinery (Figure 1).

## 2 U snRNP alterations in SMA and ALS

### 2.1 SMN

SMA is a neuromuscular syndrome that causes rapid loss of function of motor neurons until paralysis and death (Pearn, 1980). It is a major genetic cause of infant mortality and results from the homozygous inactivation of the survival motor neuron (SMN1) gene (Mishra and Mishra, 2025). In the human genome, SMN1 encodes for the SMN protein, which plays a critical role in chaperoning the assembly of the Sm core onto uridine-rich small nuclear RNA (U snRNA) precursors to generate snRNPs, the operating components of the spliceosome (Paushkin et al., 2002; Pánek et al., 2023). This protein is indispensable for cellular viability, and the loss of SMN1 is only partially compensated by the expression of its paralog, SMN2. The clinical severity of SMA is inversely correlated with the copy number of SMN2 alleles (Mishra and Mishra, 2025).

SMN is known to partner with Gemins two to eight and Unrip to form a complex that is indispensable for guiding the assembly of snRNPs. Indeed, in SMA mice and SMN-depleted cells, the levels of U snRNAs and components of U snRNPs are unbalanced, resulting in aberrant splicing (Gabanella et al., 2007; Zhang et al., 2008; Bradley and Anczuków, 2023; Ngu et al., 2025).

Loss of SMN is incompatible with life (Burghes and Beattie, 2009), and a similar lethal outcome is observed upon knockout or knockdown of other SMN–Gemins complex components, including Gemin2, Gemin3, and Gemin5 (Borg and Cauchi, 2014). This suggests that these proteins are not functionally redundant and that proper stoichiometry is essential for complex stability. Indeed, gain-of-function studies showed that Gemin2 overexpression is deleterious in yeast and flies, while increased SMN or Gemin5 levels impair viability only in a Gemin3 hypomorphic background (Borg et al., 2015). Consistently, reduced SMN levels in SMA patient-derived cells and mouse models are accompanied by decreased levels of specific Gemins (Gabanella et al., 2007; Hao et al., 2007; Zhang et al., 2008; Sapaly et al., 2020). Conversely, disruption of SMN incorporation into the complex reduces SMN protein stability (Burnett et al., 2009).

SMN levels critically determine snRNP assembly capacity (Wan et al., 2005; Boulisfane et al., 2011), which peaks in the spinal cord during neuromuscular development, coinciding with high SMN demand (Gabanella et al., 2005; Le et al., 2011; Lutz et al., 2011; Kariya et al., 2014). In SMA mouse models, reduced snRNP assembly correlates with disease severity (Gabanella et al., 2005), and restoration of this activity *via* the SMN^A111G allele rescues the phenotype (Workman et al., 2009).

Consistent with SMN’s role in snRNP biogenesis, SMN deficiency leads to widespread splicing defects. While symptomatic SMA mice show broad pre-mRNA mis-splicing (Zhang et al., 2008; Bäumer et al., 2009), early stages are characterized by splicing alterations in genes essential for motor neuron function (Zhang et al., 2013). Additional mis-spliced targets include Neurexin2 and Chondrolectin, both required for motor axon outgrowth (See et al., 2014; Sleigh et al., 2014).

It is highly likely that instead of single gene effects, inefficiencies in pre-mRNA processing consequent to severe SMN deficiency lead to a DNA damage and stress response that compromises the survival of the motor neuron (Lanfranco et al., 2017).

Currently, three pharmacological therapies for SMA are available, including nusinersen (Spinraza) representing the first FDA-approved splice-switching antisense oligonucleotide (ASO) (Crisafulli et al., 2023), Evrysdi (risdiplam), a survival motor neuron-2 (SMN2) mRNA splicing modifier, orally administered (Li, 2022) and lastly Zolgensma/Itvisma (onasemnogene abeparvovec) an AAV-based gene therapy delivering a functional copy of SMN (McMillan et al., 2025).

However, restoration of SMN levels through splicing modulation has been shown to extend beyond correction of *SMN2* exon seven inclusion and to partially rescue downstream molecular defects. For instance, Nikolai A. Naryshkin *et al.* demonstrated that treatment with small-molecule splicing modifiers significantly increased full-length SMN levels and improved motor function and survival in SMA mouse models, with transcriptomic analyses revealing a partial normalization of aberrant RNA processing and splicing alterations (Naryshkin et al., 2014). Notably, partial rescue of intron retention and associated molecular defects upon SMN restoration has been demonstrated in experimental models (Jangi et al., 2017), supporting a causal link between SMN deficiency and widespread splicing abnormalities, although full transcriptome normalization was not achieved.

### 2.2 TDP-43

ALS is a progressive neurodegenerative disorder affecting motor neurons in the brain and spinal cord and is the most common motor neuron disease (MND) in adults. The clinical features reflect the presence and location of upper or lower motor neuron degeneration at a given time. At symptom onset, the process is usually focal and then spreads to contiguous ventral spinal or brainstem regions (Talbott et al., 2016; Orrell and Guiloff, 2023; Brotman et al., 2025).

ALS is caused by mutations in superoxide dismutase 1 (SOD1) (Benatar et al., 2025), TAR DNA binding protein (TARDBP, also called TDP-43), fused in sarcoma/translocated in liposarcoma (FUS/TLS), MATR3 (Salem et al., 2022) or other genes (Tsuiji et al., 2013; Gonzalez-Perez et al., 2015). Both familial ALS caused by TARDBP mutations and sporadic ALS have distinguishing features of clinical pathology in the affected motor neurons, which include the loss of TDP-43 from the nucleus and abnormal formation of cytoplasmic aggregations containing hyper-phosphorylated and ubiquitinated TDP-43 (Neumann et al., 2006; Balendra et al., 2025).

Particularly, loss of normal TDP-43 functions and/or gain of toxic cytoplasmic aggregations could be key causative processes of sporadic ALS (Lang et al., 2024).

TDP-43 is an RNA-binding protein that predominantly localizes to the nucleus and is believed to regulate pre-mRNA splicing through binding to cis-elements in long introns and to regulate mRNA stability including its own mRNA (Sidibé et al., 2021; Duan et al., 2022). Specifically, TDP-43 and FUS localize in nuclear gems through association with the SMN complex and are involved in maintenance of the spliceosome by controlling levels of U snRNAs. Indeed, TDP-43 has been demonstrated to have a function in U snRNP biogenesis and therefore its alteration may misregulate U snRNPs and consequently may also be responsible for ALS (Tsuiji et al., 2013; Pérez-Berlanga et al., 2023). In fact, an abnormal disappearance of TDP-43 from nuclei, together with loss of Gems was observed in motor neurons from sporadic ALS patients (Tziortzouda et al., 2021).

The regulation of pre-mRNA is spatially and temporally controlled by splicing factors such as Serine/Arginine-rich (SR) proteins that concentrate in nuclear speckles, suggesting that TDP-43 localizes to nuclear speckles as well as nucleoplasm. As a matter of fact, IP- Western blotting experiments demonstrated that TDP-43 interacts with many proteins at its C-terminus including U snRNP components such as pre-mRNA processing factor 3 (PRPF3) (Tsuiji et al., 2013; Helmold et al., 2023). In ALS patients has been reported that aberrant U snRNPs accumulation was highly specific to ALS motor neurons. This abnormal nuclear accumulation of spliceosomal U snRNPs as well as snRNAs could lead to abnormal splicing in ALS motor neurons, resulting in neurodegeneration and this also suggests a possible involvement of TDP-43 in maintaining the integrity of U snRNPs.

TDP-43 can directly interact with Matrin 3, encoded by the MATR3 gene, which is a nuclear matrix RNA-binding protein involved in key aspects of nucleic acid metabolism, including chromatin organization, transcription, DNA repair, and RNA processing (Sprunger and Jackrel, 2024). Disease-associated mutations in MATR3, are linked not only with vocal cord and pharyngeal weakness with distal myopathy (VCPDM), a progressive autosomal dominant disorder characterized by distal muscle weakness, dysphagia, and dysphonia (Senderek et al., 2009), but also with ALS and TDP-43 pathophysiology (Boehringer et al., 2017). In spinal cord, Matrin three increased nuclear staining and occasional cytoplasmic localization have been reported in sporadic ALS and in patients carrying the F115C mutation (Johnson et al., 2014). Moreover, co-transfection studies report that Matrin three mutation S85C enhances Matrin three and TDP-43 interaction, increasing TDP-43 aggregation (Johnson et al., 2014; Bajc Česnik et al., 2020) and supporting a role for RNA-binding proteins in modulating TDP-43 pathology.

ALS is still an incurable and poorly treatable disease. TDP-43 and its molecular partners, being identified as central in ALS-pathogenic mechanisms (Boer et al., 2021), are currently investigated as possible therapeutic targets, with TDP-43 accumulation being effectively reduced by small interfering RNAs (siRNAs) *in vivo*, successfully leading to motor function recovery in mice (Wu et al., 2025).

## 3 Tri-snRNP alterations in retinitis pigmentosa

RP is a group of genetically and clinically heterogeneous familial blindness disorders characterized by the progressive degeneration of photoreceptors, the light-sensitive cells in the retina (Hartong et al., 2006). Several inherited mutations in genes encoding core spliceosome constituents including PRPF3 (Chakarova et al., 2002), PRPF8 (McKie et al., 2001), PRPF31 (Vithana et al., 2001), and small nuclear ribonucleoprotein U5 subunit 200 (SNRNP200) (Zhao et al., 2009; Narvaez et al., 2025) have been identified as pathogenic drivers of this group of malignancies, displaying the relevant role that splicing mechanisms play in the pathological features of retinal degeneration (Mordes et al., 2006).

### 3.1 RP-PRPF

RP-associated PRPF defects can perturb fundamental spliceosome aspects, including snRNAs stoichiometry, tri-snRNP protein composition, and spliceosome assembly kinetics, culminating in retina-specific spliceosomal dysregulation and mis-splicing of genes required for retinal homeostasis (Nottrott et al., 1999; Makarov et al., 2000; Makarova et al., 2001; Makarova et al., 2002; Schaffert et al., 2004).

Specifically, PRPF3, PRPF6, PRPF8 and PRPF31 are ubiquitously expressed protein components of the U4/U6.U5 tri-small nuclear ribonucleoprotein (tri-snRNP). In particular, PRPF8 is a core element of the spliceosome, directly stabilizing its catalytic RNA core and tuning SNRNP200 RNA helicase activity, regulating spliceosome activation (Mozaffari-Jovin et al., 2014). The functions of each PRPF gene are detailed in a previous review (Yang et al., 2021).

Unlike alterations in the PRPF31 gene, that cause protein haploinsufficiency affecting tri-snRNP assembly (Frio et al., 2008), defects in PRPF3 and PRPF8 do not affect protein expression or the tri-snRNP formation, co-expressing the mutants with their wild-type counterparts in heterozygotes (McKie et al., 2001; Chakarova et al., 2002). However, in this latter case, the spliceosomal complex has been shown to be characterized by a decreased amount of hSnu114 and hBrr2 (Tanackovic, et al., 2011a), highly involved in the catalytic activation of the spliceosome for U4/U6 snRNA unwinding (Raghunathan and Guthrie, 1998; Bartels et al., 2002), raising the hypothesis that RP-related PRPF3 and PRPF8 mutations could affect this process. In addition, in iPSC-derived retinal pigment epithelium (RPE) cells, pathogenic PRPF8 protein variants have been observed to perturb hBrr2 helicase regulation in the spliceosome, resulting in aberrant alternative splice-site selection, exon inclusion and cryptic splice sites usage and impairing spliceosome kinetics, and nuclear speckles organization (Arzalluz-Luque et al., 2021; Atkinson et al., 2024). In RPE cells, carrying a PRPF6-missense mutation reported to cause intron retention and defective spliceosome assembly (Tanackovic et al., 2011b), splicing defects were correlated with cellular and functional deficiencies, including alterations in barrier function and ciliogenesis (Liang et al., 2022). Of interest, in a mutant PRPF8 mouse model developing cerebellar neurodegeneration (Krausová et al., 2023), PRPF8 mutations seem to deregulate a cerebellar subset of circular RNAs (circRNAs), that have been largely studied in recent years as increasingly implicated in a range of neurodegenerative disorders (Lasda and Parker, 2014; Li et al., 2018). Noticeably, differential expression in circRNAs landscape has been recently confirmed also in mutant retinal organoids (Zimmann et al., 2025a), suggesting the utility of circRNAs also as potential biomarkers of retinal status and RP progression.

Such a reduction in spliceosomal function and deregulation in RNA landscape, where circRNAs could represent important contributors to eye homeostasis, may constitute a pathogenic mechanism caused by PR-PRPF mutations that co-provokes retinal dystrophy.

Gene therapy has gathered increasing attention in the context of RP. One possible approach is to revert protein haploinsufficiency by subretinally delivering functional gene copies through adeno-associated viral (AAV) vectors, a successful strategy employed by Luxturna, an AAV-based therapy for RP carrying functional copies of RPE65 gene (Ducloyer et al., 2020). Similarly, AAV-mediated gene supplementation for PRPF31, whose mutations are generally causative of haploinsufficiency, has been explored in recent studies to improve retinal function in mutant PRPF31 mouse models (Xi et al., 2022) and ciliary function in mutant PRPF31 iPSC-derived RPE cells (Xi et al., 2022). Another protein restoration approach is to exploit splice-switching antisense oligonucleotides (ASOs) aimed at re-establishing the canonical reading frame in the context of exon-skipping mutations. Following this concept, in the context of PRPF31 mutations, in patient-derived fibroblasts mRNA levels were successfully increased (Grainok et al., 2024) and a peptide conjugated ASO has been tested in a phase one study (Everett et al., 2025). Another strategy being attempted is genome editing, used to correct PRPF31 mutations in patient-derived RPE cells (Buskin et al., 2018).

### 3.2 SNRNP200

Small nuclear ribonucleoprotein 200 kDa (SNRNP200) encodes hBrr2, a spliceosomal RNA helicase whose primary role is to unwind the U4/U6 snRNA duplex to enable the structural rearrangements necessary for the catalytic activation of the spliceosome (Lauber et al., 1996). An in-depth clinical phenotypic and genetic characterization of the largest cohort of patients carrying SNRNP200 pathogenic variants has been recently published, identifying at least 21 RP-associated variants (Romo-Aguas et al., 2025).

BRR2 mutants have previously been analyzed in yeast and shown to be properly incorporated into U5 and U4/U6 snRNPs (Cvačková et al., 2014), suggesting that the assembly of the tri-snRNP complex is not affected. Nonetheless, BRR2 mutants helicase activity in unwinding U4/U6 snRNA was compromised (Zhao et al., 2009; Santos et al., 2012), and cryptic splice-site usage was promoted (Růžičková and Staněk, 2017), indicating that RP-related mutations could affect spliceosome function and activation. Indeed, BRR2 depletion *in vitro* has been reported to impair the recognition of canonical splice sites of a β-globin sequence and enhance the recognition of cryptic 5′-splice sites, underlying the relevant physiological role of BRR2 in maintaining spliceosome’s high splicing fidelity (Cvačková et al., 2014). This observation has been recently confirmed by the first successful iCLIP analysis of SNRNP200, that strongly associates SNRNP200 mutations with aberrant contacts with its substrates and the impairment of its helicase activity (Zimmann et al., 2025b). Overall, these findings underline the relevant physiological role of BRR2 in maintaining spliceosome’s high splicing fidelity, especially in a tissue like the retina, with such high transcriptional levels (Li et al., 2014), where even a slight decrease in splicing fidelity could lead to the buildup of mis-spliced mRNAs, ultimately causing photoreceptor cells death.

Despite its pathological relevance in RP and being already studied as a therapeutic target in cancer (Baumann et al., 2024; Yang et al., 2024), there are currently no available SNRNP200-specific clinical therapies for RP, and research is focusing on SNRNP200 genetic, molecular and mechanical characterization.

## 4 Discussion

This review highlights spliceosomopathies as a group of disorders caused by pathogenic variants in core spliceosomal components and RNA-binding proteins, with prominent effects on the nervous system (Griffin and Saint-Jeannet, 2020; Ayers et al., 2023; Bou-Rouphael et al., 2025; LeBlanc et al., 2025; Nava et al., 2025). Despite the ubiquitous expression of the spliceosome, its dysfunction leads to marked tissue-specific vulnerability, particularly in the retina and spinal cord motor neurons, as exemplified by retinitis pigmentosa, spinal muscular atrophy, and amyotrophic lateral sclerosis (Griffin and Saint-Jeannet, 2020). Across these conditions, defects in snRNP biogenesis, spliceosome assembly, and splicing fidelity emerge as shared pathogenic mechanisms driving RNA mis-processing and cellular dysfunction.

Altered RNA metabolism is now linked to more than 30 human diseases, including neurodevelopmental, neurodegenerative, hematological, and craniofacial disorders, and the number of disease-associated spliceosomal variants continues to expand (Griffin and Saint-Jeannet, 2020; Griffin, 2025). In SMA and FUS- or TDP-43–linked ALS, impaired U snRNP homeostasis and biogenesis represent central disease mechanisms, whereas in retinitis pigmentosa, mutations in PRPF proteins and SNRNP200 primarily affect tri-snRNP composition, spliceosome activation, and splice-site selection. These observations illustrate how distinct perturbations of the same core machinery can result in divergent clinical phenotypes. Even though current research on SMA and TDP-43-related ALS is more focused on snRNP assembly disruptions or modifications rather than on U snRNAs, it is relevant to emphasize that also RNA spliceosome components may deserve attention in these pathologies. Recently, four pathogenic mutations in RNU4-2, encoding U4 snRNA, and in four paralogs of the U6 snRNA have been identified as novel causes of RP, interacting with factors like PRPF8 and 31 and impairing snRNP biogenesis, broadening perspectives on new research topics and new molecular therapeutic targets (Quinodoz et al., 2026).

A major unresolved issue is the basis of tissue specificity. Differential expression of spliceosomal components and their interactors may contribute to selective vulnerability, but this alone is unlikely to fully explain disease patterns. Tissues such as the retina and brain display high transcriptional activity and extensive alternative splicing, rendering them particularly sensitive to defects in splicing fidelity (Bäumer et al., 2009; Aísa-Marín et al., 2021). However, the lack of retinal involvement in some spliceosomopathies indicates that additional factors, such as unique nuclear architecture, the functional consequences of altered transcript diversity, and potential non-canonical roles of spliceosomal proteins, must be considered. In addition, aging must be taken into account. Despite being one of the most prominent risk factors for neurodegeneration, age-related neuronal degradation mechanisms are several and are still under investigation. Aging is sufficient to cause TDP-43 mislocalization (Rhine et al., 2025) and generate chronic stress granules whose composition is different from canonical stress granules, lacking key RNA binding proteins for RNA splicing and transport (Rhine et al., 2025). Splicing inaccuracy during aging is common across all human tissues, but in brain it is more impactful due to alternative splicing relevance and the higher need of RNA binding proteins, affecting genes involved in neuronal function (García-Ruiz et al., 2025). Finally, it is known that a plethora of regulatory RNA molecules is constitutively encoded in the human body, and that their balance has a big role in the maintenance of cell homeostasis. For example, disruption of the U1/vU1 snRNA ratio is associated with the neuropathology of SMA disease (Vazquez-Arango et al., 2016), and in cases like this compensatory mechanisms involving non-canonical U-snRNAs occur resetting the physiological balance (Vazquez-Arango et al., 2016). During aging, these mechanisms decline. Indeed, understanding inaccurate splicing processes and decline of compensatory mechanisms during aging could help comprehend better how neurons are driven towards neurodegeneration.

Although the molecular mechanisms underlying spliceosomopathies remain incompletely understood, gene-based therapies are emerging as the most promising therapeutic strategies (Lonfat et al., 2025; Xie et al., 2025) Current approaches include gene replacement and enhancement, splice-switching antisense oligonucleotides, and genome editing. Further progress will require a deeper understanding of tissue-specific genetics and genotype–phenotype correlations, the development of robust disease models, and a rigorous functional characterization of pathogenic variants to enable effective, mechanism-based therapeutic interventions.
